# AI in Radiation Oncology: A Comprehensive Review of Current Applications and Future Directions

**DOI:** 10.7759/cureus.92964

**Published:** 2025-09-22

**Authors:** Fabeha Zafar, Jessica Vilsan, Shinjit Mani, Ali R Al Yousif, Sandra E Cano-Reyes, Godwin Abraham, Joao F de Barros Neto, Bashir Imam, Moyosoreoluwa Aluko, Soumyadeep Sikdar

**Affiliations:** 1 Department of Internal Medicine, Dow University of Health Sciences, Karachi, PAK; 2 Department of Internal Medicine, Maharashtra University of Health Sciences, Nashik, IND; 3 Department of Medical Oncology, Chittaranjan National Cancer Institute, Kolkata, IND; 4 Department of General Surgery, University of Basrah, Basrah, IRQ; 5 Department of Pediatrics, Hospital Pediátrico La Misericordia, Bogotá, COL; 6 Department of Oncology, Midland Metropolitan University Hospital, Birmingham, GBR; 7 Department of Internal Medicine, Universidade do Estado do Rio de Janeiro, Rio de Janeiro, BRA; 8 Department of Internal Medicine, Jackson Park Hospital and Medical Center, Chicago, USA; 9 Department of Internal Medicine, Babcock University, Ilishan-Remo, NGA; 10 Institute of Medical Sciences, Banaras Hindu University, Varanasi, IND

**Keywords:** ai, artificial intelligence, machine learning in radiology, radiation oncology, radiotherapy

## Abstract

At its core, radiation oncology uses knowledge and expertise from multiple precise disciplines such as physics, mathematics, and computer science, which converge with biology and medicine. This is why the rapidly developing AI use in medicine has immense potential in radiotherapy at different levels, such as image reconstruction, volumetric segmentation, radiotherapy delivery, and treatment response. In this review, we aim to provide a summary of current AI use in radiation oncology, mapping in which areas these tools have already been incorporated, as well as their contributions to radiotherapy workflow. Here, we analyze how machine learning software increases the efficiency and accuracy of radiation treatment planning, delivery, and outcome prediction, providing a comprehensive picture of the advancements, limitations, and future directions of AI use in radiotherapy. The radiotherapy workflow consists of multiple intensive steps that are crucial to planning individualized treatment. The introduction of AI assures quality and standardization and reduces variability and time spent in processes such as image reconstruction, segmentation, and dose calculation. Deep learning segmentation reduces planning and delivery time without sacrificing quality. AI predictive capabilities enable clinicians to anticipate and reduce treatment-related toxicities through accuracy based on clinical parameters and image data. Building powerful models requires extensive and robust high-quality data that maintains privacy and HIPAA compliance and must be collected with precision and accuracy. This process, however, can present ethical and logistical obstacles, such as clinical validation needs and reproducibility standards that must be addressed to fully integrate AI into clinical workflows alongside human oversight.

## Introduction and background

The term “AI” refers to programming computers to simulate human-like reasoning; it has evolved into more sophisticated systems, such as deep learning (DL), that mimic the brain’s information processing [1]. AI is rapidly expanding in terms of its depth and breadth of use, and it has become a transformative global force with potential implications for health care [2]. The future of AI heralds unprecedented change for the field of radiation oncology, where it is poised to revolutionize patient care and clinical workflows. AI is seen as a great tool that could “think and act humanely without losing its rationality.”

Radiation therapy is a multistep, complex, and labor-intensive process requiring precise planning and application. The radiation therapy workflow involves several steps, including initial treatment decision-making, simulation, treatment planning and preparation, quality assurance (QA), delivery of radiation, and posttreatment follow-up care [3]. Various components of this workflow are being automated using AI. These include image reconstruction, target and tissue segmentation, treatment planning, radiotherapy delivery, and treatment response assessment [4]. This whole process is heavily reliant on technological infrastructure such as CTs, MRIs, imaging reconstruction software, and linear accelerators, which makes the introduction of AI in radiation oncology practice smoother and consequently faster than in other medical specialties [5]. Additionally, this technological equipment generates comprehensive datasets that require further analysis by a team composed of medical physicists, dosimetrists, radiation oncologists, and radiation therapists, decreasing the efficiency of the workflow. Automation in radiotherapy can not only lessen the workload but also reduce the long delays that would have otherwise impeded the swift initiation of proper management in an already anxious patient.

Given the growing burden of health care staff shortages and an aging population, AI’s ability to screen large amounts of data with increased accuracy and speed, at a fraction of the cost of human staff, makes its integration not just advantageous but essential. Its applications, such as image processing, 3D structure contouring, radiation therapy administration, and response assessment, are already being developed. AI is being used and tested in various applications at nearly every stage of this workflow. By doing so, we can determine and deliver to patients a treatment course that is faster, more precise, and more affordable, thus reducing waiting times, improving efficacy, and decreasing side effects [5,6]. AI can be integrated throughout the radiotherapy workflow, from image processing (including image registration and segmentation [5]) to treatment planning, as well as after the administration of radiation therapy, in predicting radiation-related aftereffects (namely radiation pneumonitis [7] and radiation-induced temporal lobe injury [8]), resulting in an overall efficient and personalized workflow. With the rapid development of AI in health care, radiation oncology holds great promise, with several applications being reported in medical literature, and some are already being implemented in clinical practice in steps of planning, segmentation, and delivery of radiation [9].

This narrative review compiles the advancements over the last 10 years and explores the integration of AI in the field of radiation oncology, and it aims to provide our readers with a holistic view of its current and future clinical applications. It specifically addresses the use of AI in automated tumor contouring, image segmentation, dose prediction, treatment planning, and outcome prediction [10,11]. The studies in focus were those that demonstrated clinical relevance, meaning that they used real patient data and were tested in actual clinical settings, showed an impact on diagnosis, treatment, or patient care, and highlighted both the benefits and limitations of using AI. However, the review does not go into the technical details and algorithm design of AI models. The goal is to provide an overview of key advancements, current challenges, and future directions of meaningful integration of AI in radiation oncology.

The potential future of AI in radiation oncology lies in the ability to strengthen physician-AI collaboration. It also allows physicians to be more efficient with less time spent on planning tasks and more time spent with patients [9]. More work and effort are required in several directions to fulfill the urgent need to effectively promote the safe use of AI and strengthen its future application [6].

Challenges remain, including the need for large data collections, interpretation, and more reliable and effective models. A lack of knowledge among clinicians about the advantages of incorporating AI into their practice is another challenge. A comprehensive education plan needs to be incorporated to train physicians on AI to decrease the knowledge gap and increase trust among clinicians. In addition, peer-to-peer education can further help increase knowledge, confidence, and acceptance of AI [5].

## Review

Machine learning in the radiotherapy workflow

**Figure 1:**
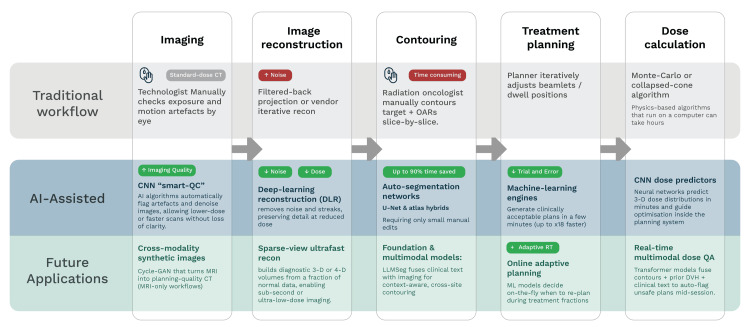
Comparison of workflow in radiotherapy, traditional workflow vs. AI-assisted workflow [5,9-15] Original illustration created by co-author Ali R. Al Yousif

The initial integration of AI in the field of radiation oncology has already begun in the clinical setting but still has much more potential for growth and to improve the overall efficiency of the workflow [9]. The radiotherapy workflow consists of multiple intensive steps, each of which is crucial for planning an individualized treatment. This multistep process generally requires a significant amount of time, resources, and staff and traditionally involves a radiation oncologist manually delineating the tumor and organs at risk (OARs) on CT images, which can be incredibly time-consuming; for instance, contouring six OARs in head and neck cases can take up to 108 minutes [12]. Furthermore, this manual process often leads to variability among clinicians. This interobserver variation has been well-documented and is a critical issue that can cause inaccuracy in treatment [13]. The introduction of AI alters this multistep process, creating a more streamlined, AI-assisted workflow, while promising future applications with further improvements (Figure 1).

AI utilizes neural networks to recognize objects in a scan (convolutional neural networks (CNNs)) or to memorize them (recurrent neural networks) [3]. Applying these neural networks to perform certain tasks is what comprises the field of DL, ultimately resulting in superior pattern recognition, which is what is incorporated in machine learning and ultimately AI as a whole.

With the rapid evolution of AI, treatment planning steps were the first to be considered for automation using these technologies. DL-based technologies were favored because tasks like tissue segmentation and auto-contouring rely heavily on automated image recognition. CNNs and U-Net architecture have been widely used for the past decade for this purpose with great success [14]. In one instance, a study conducted by Urago et al. showed a significant reduction in time required to complete the delineation process by automatic delineation, which took six minutes when compared with the three hours required for manual delineation for head and neck cancers. Although promising, this study also comes with a few uncertainties regarding accuracy and limited datasets [13]. In a survey of French radiation oncologists, 35% of the participants saved between 50% and 100% of the segmentation time for OARs using AI segmentation software [15].

The introduction of AI into the field is backed by the assurance of quality and standardization to reduce variability and time spent [16] and can be implemented at various stages of the radiotherapy workflow, some of which are described below.

Image Acquisition

Imaging is essential right from the beginning when planning the treatment and is utilized even after the treatment to monitor the progress. CT is the preferred mode of imaging due to dose calculation compatibility and is used at the start of the planning process to delineate the area of interest, including the tumors and the surrounding critical structures. This makes the image reconstruction process, which is usually performed by dedicated computers in the background, crucial. Although highly efficient and stable, there is a possibility of increased artifacts and noise in undersampled and noisy sensor measurements during computerized image reconstruction. Therefore, to enhance the quality of the resulting image and to reduce the time taken to reconstruct it, multiple DL-based image reconstruction methods have been introduced. This can be achieved using multiple DL-based methods, including post-reconstruction denoising of low-dose CT images [5]. In the article by Fu et al., multiple studies were described utilizing different systems, including deep CNNs, which were used to suppress noise; a residual encoder-decoder CNN to improve low-dose image quality; and a generative adversarial network for denoising. These come with their own set of drawbacks, one of which is that they need both a low-dose CT image as well as its high-quality version, which, practically speaking, may not be available. Another issue that post-reconstruction quality enhancement faces is that it may result in blurring of edges in the final image [5]. It has also been proposed that DL methods can be combined with the filtered back projection, an analytical reconstruction method, to attain more precise reconstructions even with truncated data [5].

Image Contouring

Delineating and contouring targets and OARs manually has always been cumbersome, vulnerable to human error, and prone to human variations due to the subjective nature of the process [17]. The operation of AI segmentation has been tested on multiple treatment sites, including head and neck, prostate, esophagus, and breast tissue cancers. Although better at contouring of OARs, AI faces challenges when it comes to target contouring due to the multimodal imaging requirements for target segmentation and variations in the target itself when considering tumor control over toxicity. In addition to reducing the time taken for contouring, AI is also expected to reduce interobserver variations and improve its overall utility depending on training data, model design, and loss functions (mean square error leading to blurring). In a study conducted by Jiang et al., organ segmentation-based AI dose mapping (AIDA) was applied to the esophagus and the heart and evaluated. The analysis showed that AIDA produced similar estimates of dose to manual DA [18]. The accuracy of atlas and AI-based delineation of OARs in patients with prostate and head and neck cancers was compared to manual delineations in a study conducted by Urago et al. and was found to have comparable efficiency, with AI-based segmentation requiring less manual correction than atlas-based segmentation [13]. In a study by Xu et al., an asymmetric multitask attention network (AMTA-Net) was introduced, which outperformed traditional methods in segmenting the prostate bed post-prostatectomy [19]. The efficiency of complex TMI/TMLI treatment planning can be significantly improved by AI auto-segmentation, achieving ~75% manual workload reduction across most structures, as demonstrated by a study conducted by Watkins et al. [20]. Technologies that traditionally relied on CNNs or machine learning started gaining adoption around 2017-2018 [21] and have subsequently been heavily researched and proven to achieve significant improvements in accuracy, speed, and interobserver variability [22].

Recent technologies have emerged that promise even more improvements. One of them is an approach based on the Segment Anything Model (SAM) by Meta, which offers live human interaction, prompt functionality, and the ability to handle diverse tasks and anatomical structures. Using simple box prompts, researchers were able to produce clinically acceptable segmentations with SAM on CT images of multiple anatomical sites (prostate, lung, gastrointestinal, and head and neck) without any additional training on medical images [23].

Image Synthesis

One of the most important sources of data when planning and decision-making in a radiotherapy workflow is medical images. Depending on the site of interest and the properties to investigate, some imaging modalities are more suitable than others, and there is a conventional need for the use of multiple modalities to completely grasp the intricacies of an individual's anatomy and the tumor tissue.

**Figure 2:**
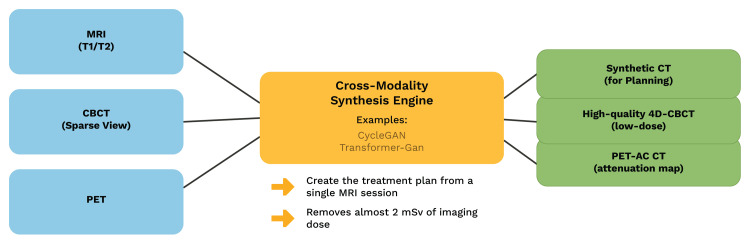
Cross-modality image synthesis in radiotherapy [5] Original illustration created by co-author Ali R. Al Yousif

In a novel approach, generative modelling is used to translate across different modalities using the data distribution of each modality. In this regard, there is much promise shown by GAN-based AI. Implementation of this approach is potentially cost-effective and leads to reduced patient exposure to excess radiation as a direct result of the reduction in recurrent imaging scans, which are usually needed [5]. Although GAN-based cross-modality synthesis shows promise, further studies still need to be done to establish its value. This technique of generating a synthetic image of one modality from another is known as cross-modality image synthesis (Figure 2).

Treatment Planning and Dose Calculation

Dose planning requires frequent parameter adjustments and calculations. Similarly, adaptive radiotherapy (ART) requires continuous calculations throughout the treatment to achieve the best results. Various DL methods have been used for complex dose calculations, such as in volumetric modulated arc therapy (VMAT) on MR-Linac systems [24]. In another instance, a significant reduction in mean planning time (2.38 minutes vs. 43.13 minutes for conventional techniques) was achieved using a machine learning-based planning workflow for prostate low-dose-rate (LDR) brachytherapy [25]. Although showing a massive reduction in mean planning time, the results of this study may not be generalizable across all institutions, and further studies must be conducted to assess the general applicability. Another striking example comes from Lin et al., who developed an AI-assisted pipeline for hippocampal avoidance whole brain radiotherapy (HA-WBRT) [26]. Traditionally, contouring brain structures and generating treatment plans for HA-WBRT is a labor-intensive and time-consuming process. However, their DL-based system was able to complete clinically acceptable plans in just 10 minutes per patient, all while maintaining high quality. This highlights AI’s capacity to streamline even the most complex planning tasks. Bolten et al. validated a fully automated workflow for prostate cancer treatment planning. Although the sample size was small, the AI-generated contours closely matched expert delineations, with acceptable dose coverage and low interobserver variability [27]. Tsekas et al. demonstrated how DL could be integrated into dose calculation for VMAT using MR-Linac systems [24]. Their model accurately predicted radiation dose distributions across 17 new plans, suggesting that AI can make dose calculation both faster and more precise.

LLM-driven models provide a new feature that is game-changing for dose planning and multimodality. In terms of technological innovation, the development of multimodal AI systems is pushing the boundaries even further. Oh et al. introduced LLMSeg, a large language model-driven tool that integrates clinical text with imaging data to enhance target volume contouring [28]. Applied to breast and prostate cancer datasets, LLMSeg significantly outperformed image-only models, demonstrating improved accuracy, generalization, and data efficiency. By leveraging multimodal AI, models like LLMSeg have demonstrated the ability to integrate both clinical information and CT images for clinical target volume (CTV) delineation [28]. This approach underscores the potential of combining clinical context with image analysis to support more nuanced and personalized treatment planning.

Use of AI to increase precision and efficiency

Enhancing Precision in Radiotherapy Delivery

In radiotherapy, precision depends on the accuracy and consistency with which radiation is delivered to the tumor while sparing healthy surrounding tissues. It ensures that the intended dose hits the target exactly, minimizing side effects and maximizing tumor control. In clinical practice, this means accurate tumor and OAR delineation, dose calculation, and treatment delivery. Traditionally, achieving this level of precision has involved significant reliance on clinician expertise, with manual tasks contributing to variability in treatment planning. With the introduction of AI into the radiotherapy workflow, new approaches have been designed to address these issues.

AI for Workflow Efficiency and Time Reduction

In the field of radiation oncology, efficiency refers to the ability to complete complex clinical processes, such as treatment planning, contouring, and dose delivery, in a timely and resource-conscious manner without compromising the quality of care. This means minimizing the time spent on manual tasks, reducing clinician workload, and ensuring that patients can begin their treatment as soon as possible. Increasing efficiency is especially crucial in high-volume cancer centers and in low-resource settings, where staff shortages and technological limitations often lead to delays.

One of the key reasons to use AI in radiation oncology is to improve the efficiency of RT workflow, to decrease the time required, and to improve accuracy. By leveraging advanced algorithms, AI can standardize how OARs and tumors are defined across different patients and institutions. There have been attempts to develop AI-based models to automate either the full process or a part of it to improve efficiency and accuracy. While some tried to develop their AI system for the required task, others used and evaluated commercial AI software for the same.

Clinical Validation of AI-Based Systems

Lin et al. developed an AI-assisted system that is capable of automatically segmenting brain structures, including the hippocampus, to develop treatment planning for HA-WBRT. All their AI-generated plans adhere to the Radiation Therapy Oncology Group (RTOG) 0933 criteria and could produce a plan within 10 minutes for each patient, thus reducing the time needed while improving efficiency [26]. In another experiment, Nicolae et al. investigated a machine learning-based prostate implant planning algorithm (PIPA) system against a conventional system. They demonstrated that the PIPA arm had significantly less planning time (2.38 ± 0.96 minutes) compared to the conventional technique (43.13 ± 58.70 minutes), while producing noninferior postoperative dosimetry to that of an expert [25]. Similarly, Xia et al. also developed an AI-based full-process solution for rectal cancer radiotherapy. They found no significant differences between the automatically generated plans and manual plans [29].

Commercial AI Tools in Practice

Commercially available AI-based programs have also been tested and found to be precise enough to improve accuracy and efficiency. A commercial AI-based auto-contouring application was tested by Hoque et al. Their study included a database of 40 patients treated with radiation therapy, among whom 20 patients were treated for prostate cancer and 20 patients were treated for head and neck cancer. The auto-contouring system was applied retrospectively to assess geometric accuracy and the influence on optimized dose. They concluded that the time required for the RT workflow is significantly reduced without affecting dose distribution and plan quality when an auto-contouring system is used in combination with human review [30]. In another study, NeuroQuant, an FDA-approved software that provides segmentations of brain structures, was tested for automated hippocampal segmentation and treatment planning to generate HA-WBRT plans, which are clinically acceptable. T1 post-contrast brain MRIs of 100 adult patients, who were treated for brain metastases outside the hippocampal region, were processed by the program to generate segmentation images. In total, 99 out of 100 cases had acceptable hippocampal segmentations, which did not require manual intervention [31]. The program known as Limbus Contour, Version 1.5.0 (Limbus AI Inc., Regina, SK, Canada), a deep CNN model based on a U-Net architecture, also went through testing. Both manual and automatic segmentation were compared on disease-specific OARs (head and neck, prostate, breast, and rectum). Multiple parameters were evaluated, including contouring time, volume variation, and the Dice similarity coefficient (DSC). The results reported up to 65% time savings across multiple tumor sites, including head and neck, breast, prostate, and rectum, which simplified the workflow and reduced interobserver variability [32]. Another commercial AI software, Organs-RT, was found to have an impact in minimizing overall contouring time and increasing efficiency in radiotherapy treatment planning. The OARs generated by Organs-RT for the bladder, heart, lungs, and femoral heads had an overall DSC ≥ 0.92. This study also measured time efficiency. Sending 10 pelvic cases for contouring by Organs-RT saved 6.6 hours, sending 10 thoracic cases saved 5.4 hours, and sending 10 head and neck cases saved, on average, 3.2 hours, indicating the reliability of this auto-contouring, which decreases overall contouring time and improves efficiency in radiotherapy treatment planning [33]. In a novel attempt, Doolan et al. compared the OAR contours generated by five commercial AI auto-segmentation solutions (Mirada, MVision, Radformation, RayStation, and TheraPanacea) against manually created expert contours from 20 head and neck, 20 lung, 20 breast, and 20 prostate patients. Out of these five commercial tools, Mirada, MVision, and RayStation have FDA 510(k) clearance; Radformation has CE European approval; and TheraPanacea has both CE and FDA approval. Volumetric and surface DSC (vDSC and sDSC), Hausdorff distance (HD), and added path length were used for comparison, along with the time taken for completion of the work done by manual mode and by AI. All five commercial AI solutions successfully prepared high-quality contours in a significantly reduced time compared to manual contouring [34].

Future Directions: Adaptive Planning and QA

In a similar attempt, in a study by Meixner et al., while validating commercially available ESTRO guideline-based AI auto-segmentation models, the RayStation treatment planning system (Model 1), an auto-segmentation software based on guideline-based DL MVision (Model 3), demonstrated high-quality, accurate, standardized, and efficient target volume contouring [35]. Although clinical accuracy is a concerning factor, a study conducted by Strolin et al. showed that a commercial AI-based contouring tool reduced time spent contouring by up to 92%, maintaining high levels of clinician satisfaction [36]. Similarly, Turcas et al. validated a commercially available DL segmentation tool for brain OARs using MRI, demonstrating substantial reductions in contouring time, down from 20 minutes to just over one minute, without compromising dosimetric accuracy in large structures [37].

The incredible show of efficiency is especially evident when applied in low- to middle-income countries (LMICs), as pointed out in the study by Kibudde et al., which reported that AI-based auto-segmentation reduced contouring time from around 60 minutes to just two minutes per case [38]. This translates to an annual savings of roughly 1,000 clinician hours that could be redirected to patient care. Although challenges like limited internet infrastructure in some LMICs remain, the efficiency gains offered by AI are undeniable.

Wider reviews and systematic analyses have further confirmed these benefits. For example, Fu et al. and Hoebers et al. noted that AI tools can reduce time spent on contouring and planning by up to 90% in certain settings. Beyond speed, AI improves consistency in organ delineation and reduces interobserver variability, which is crucial for ensuring high-quality, reproducible care [5,9].

DL is a subset of machine learning that uses deep neural networks (artificial neural networks with multiple layers) to analyze data. DL-based auto-segmentation models have emerged as a promising solution. It has been tested and found to be highly accurate and efficient compared to manual contouring [39]. Gibbons et al. compared the accuracy of DL and atlas-based auto-segmentation with clinical “gold standard” reference contours. They concluded that DL segmentation broadly outperformed the atlas-based contouring for head and neck and pelvic OAR contours with statistically significant median DSC improvements in parotids (0.80 atlas/0.87 DL), mandible (0.91 atlas/0.94 DL), submandibular glands (0.68 atlas/0.80 DL), rectum (0.77 atlas/0.87 DL), bladder (0.88 atlas/0.96 DL), and left and right femoral heads (0.96 atlas/0.98 DL). The DL-based program was significantly faster when compared with the atlas-based solution as well as manual contouring; for instance, the contouring of the left parotid by DL took 0.7 minutes, atlas took 2.1 minutes, and manual contouring took 2.5 minutes [40]. Similar results were achieved when Ma et al. developed a DL tool based on VB-Net architecture to delineate CTVs of the pelvic lymph drainage area and parametrial area. They concluded that the accuracy of the DL-based tool was comparable to that of experienced senior radiation oncologists. This study also displayed mean DSC increases: 0.20 for dCTV2 (parametrial region) and 0.03 for pCTV1 (pelvic lymph drainage area); it also demonstrated a reduction in mean contouring time (9.8 minutes for dCTV2 and 28.9 minutes for pCTV1). A DL-based program for auto-segmentation improves contouring accuracy and clinical efficiency by reducing contouring time [7].

A specific algorithm of DL is the U-Net, which is being tested in the field of radiotherapy. He et al. developed and internally tested a U-Net-based framework known as the nasopharyngeal carcinoma diagnosis segmentation network framework (NPC-SDNet). Notably, it outperformed clinicians and achieved a diagnostic accuracy of 94.0% while processing 1,000 frames per minute. This specific model not only demonstrated excellent real-time diagnostic and segmentation accuracy but also offered a promising tool to enhance the precision of nasopharyngeal carcinoma diagnosis [41]. Similarly, an attention-based U-Net for parotid tumor auto-segmentation was created by Xia et al. on the MRI T1w, T2, and T1wC images of 285 patients diagnosed with parotid tumors. The outcome indicated that the performance of this model is efficient enough to be comparable to the manual segmentation done by radiologists [42]. Nie et al. developed and trained a system, namely SagNet, for automatic contouring of the CTV and OARs in cervical cancer patients using retrospective data of 203 patients. This system brought down the manual revision time for automatic CTVs to 9.54 ± 2.42 minutes relative to the fully manual delineation time of 30.95 ± 15.24 minutes, improving the performance and efficiency of the delineation process [43]. In a similar manner, Zhong et al. trained and validated a U-Net-based full CNN for the automatic delineation of OARs of head and neck cancers by using annotated CT images of 364 clinical HNC patients. They measured automated delineation accuracy using the DSC and 95% HD and assessed efficiency using a questionnaire. They concluded that auto-contouring significantly shortened the manual delineation time from hours to minutes and achieved a clinical acceptance level similar to manual delineations [44]. Apart from all these, an experiment was done by Zhang et al., and their model, proposed as the layer-volume parallel attention-U-Net model, based on a 2D-3D architecture, outperformed eight typical models [45]. Similarly, Kazemimoghadam et al. proposed a saliency-guided DL model (SDL-Seg) to automatically delineate tumor bed volumes in postoperative breast cancer patients. Their model was trained by application of four-fold cross-validation and a dataset with 145 CT images from 29 patients and outperformed traditional U-Net architectures, improving Dice similarity scores by 13% while maintaining low computational costs [46].

Nicolae et al. compared conventional manual planning with a machine learning-based approach for LDR brachytherapy. They found that the AI system significantly reduced planning time without compromising the quality of treatment. Moreover, because machine learning models are inherently consistent, they offer the added benefit of standardizing treatment planning across institutions and clinicians [25].

AI’s promise is not just theoretical; it has been tested and implemented in real clinical environments. The application of AI extends across various anatomical sites. For instance, Kakkos et al. demonstrated how AI can be used for parotid gland segmentation, ART, and multimodal CT-CBCT image registration [47]. Belue et al. developed a 3D CNN to segment the prostatic urethra on non-catheterized MRI, supporting more accurate planning for prostate cancer patients [48].

In ART, where plans must be frequently updated to account for anatomical changes, AI also shows potential. Mastella et al. reviewed AI applications in CT-based adaptive planning for head and neck cancer. They found that AI could automate many time-intensive steps, such as synthetic CT generation and auto-segmentation, while maintaining high performance [49]. AI is also proving invaluable in QA within radiotherapy. Chan et al. reviewed how machine learning models can predict QA outcomes for intensity-modulated radiotherapy and VMAT, based on system performance metrics. Their findings support AI’s role in early anomaly detection and suggest a future where much of QA could be automated, thereby streamlining workflows and improving reliability [50].

AI is emerging as a transformative force in improving radiotherapy workflows. By automating repetitive tasks, accelerating complex calculations, and enhancing decision-making through data integration, AI offers a powerful solution to the inefficiencies that currently burden radiation oncology.

Use of AI in risk stratification and predicting radiotherapy outcome

Beyond automation, AI’s predictive capabilities are enabling clinicians to anticipate and mitigate treatment-related toxicities. In radiation oncology, accurate risk stratification is particularly important for predicting outcomes, minimizing side effects, and personalizing treatment plans. With the integration of AI in radiotherapy, this predictive accuracy has substantially increased, which in turn has assisted clinicians in decision-making and treatment planning.

The response to radiotherapy varies among patients; therefore, to optimize treatment plans, it is critical to predict individual responses. AI-based algorithms are promising in predicting tumor responses based on clinical parameters and imaging data. For example, DL models, trained on CT, MRI, or PET scans, can help in predicting response by detecting early changes in tumor size and shape during treatment [51].

Another example is the use of CNNs to analyze pretreatment imaging data. These networks have been trained to differentiate between responders and nonresponders to treatment, even before clinical signs are visible [52]. This allows clinicians to adjust treatments to improve outcomes and minimize side effects by increasing the intensity of treatment for responders, who can benefit from higher doses, and by reducing the intensity for nonresponders to avoid unnecessary adverse effects.

One of the most concerning adverse effects of radiotherapy is organ-specific toxicities. Developments in AI have proven to be beneficial in this aspect as well. Models have been used to predict radiation-induced lung toxicity by analyzing the distribution of radiation doses to lung tissues, which is represented by dose-volume histograms [53]. AI and CT imaging features were used to predict normal lung dose during radiotherapy for breast cancer, as evidenced by a multicentric study conducted by Ma et al., and their model could accurately forecast lung radiation exposure that in turn predicted the risk of radiation-induced pneumonitis [7]. In a similar vein, Quan et al. employed an XGBoost machine learning model combining radiomics and dosiomics to predict radiation-induced hypothyroidism in patients undergoing tomotherapy for nasopharyngeal carcinoma [54]. Another study made use of deep neural networks to predict side effects following breast cancer radiotherapy and achieved 90% accuracy in predicting cardiotoxicity in the patients posttreatment [55]. Newer studies suggest that AI can also identify which specific tissues or organs are most likely at risk to suffer damage. One such study highlighted how AI models can be utilized to predict the risk of esophagitis in patients undergoing thoracic radiation therapy; the model was successful with an accuracy rate above 80% [56]. The capacity of AI to forecast toxicity extends to other cancer types as well. Ladbury et al. used explainable AI in a secondary analysis of the RTOG 0617 trial to identify dosimetric predictors of high-grade pulmonary and esophageal toxicities in non-small cell lung cancer. Their machine learning models not only validated existing dosimetric thresholds but also outperformed logistic regression [57]. These studies highlight the potential for AI to refine safety margins and enable proactive risk-adjusted treatment planning.

Radiation therapy can potentially lead to many side effects, including nausea, fatigue, and skin irritation. In more severe cases, it can lead to long-term complications such as organ fibrosis and toxicity. Therefore, minimizing these side effects by predicting them beforehand is essential for improving treatment outcomes and patients’ quality of life. AI-based models have been trained to predict the likelihood of these side effects. For instance, radiomic features, which are data derived from medical images, including texture and heterogeneity of tissues in treatment areas, can predict the risk of skin reactions and mucositis in head and neck cancer patients [58].

In summary, AI offers powerful tools for radiotherapy outcome prediction and risk stratification. Use of AI in radiation oncology can lead to more personalized treatment, better efficiency, and improved patient safety. However, to fully incorporate AI in routine clinical practice, future research and testing are required. Additionally, its integration into existing workflows will require coordinated, multi-institutional collaboration.

Need for a large dataset to feed the AI algorithm for producing accurate results

Machine learning algorithms use computational methods to “learn” information directly from data [50]. In this sense, the implementation of AI in the different radiation therapy steps creates the need to feed the models with high-quality data to learn from. Such data must ideally be robust and reliable and represent the population. In order to achieve the above, a detailed inclusion and exclusion criteria list is needed to extract data from institutional archives, such as picture archival and communication systems, treatment planning systems, and electronic hospital records [9].

Data learning can be achieved in a supervised or unsupervised fashion, depending on whether or not the data includes the desired outputs and their correlation with the input variables [50]. In medicine and radiation therapy, supervised learning is more frequently and widely used.

**Figure 3:**
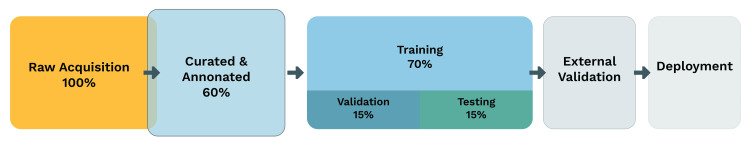
Radiotherapy AI data pipeline [9,50,59,60] Original illustration created by co-author Ali R. Al Yousif

The data is typically divided into the following three subsets: the first one for training of the AI algorithm, the second for tuning (often called validation), and a third, independent testing subset for external validation. Some common ratios for splitting the data are 60:20:20, 70:15:15, or 80:10:10 [59]. It is critical to ensure there is no overlap between these sets to maintain model integrity and measure generalizability [9,60]. The main purpose of using the testing dataset is to validate the generalizability of a trained model [50]. The necessity for large datasets becomes evident when considering these multiple stages (Figure 3).

Transitioning into standardized AI-assisted radiotherapy requires large datasets in order to train the models and submit them to internal and external validation. With the current health system still evolving into digital medical recording, a unified registry of patient information is not yet attainable to feed the models. These elements directly impact the performance and reliability of a model [60]. Data acquisition is, therefore, a challenge that limits the interoperability of models between institutions.

Ideally, differences in data availability, quality, annotation, and follow-up that might lead to a disadvantage in representativeness for a certain subgroup of patients should be minimized [9]. An AI specialty called natural language processing has been used to automate unstructured data from clinical notes in an attempt to favor more robust dataset building [61].

Building larger datasets promotes heterogeneity within AI models, which hopefully would include data from patients within different demographics with different types, locations, and stages of cancer [62]. There is a long road in front of us, especially in the prospective validation of DL models, and multicentric clinical trials are needed to assess the impact of using AI algorithms in radiotherapy [61].

One of the main issues regarding data compilation and processing is privacy assurance. Feeding AI algorithms with patient medical information comes with the risk of breaching confidentiality [62]. Hence, efforts need to be made to preserve the anonymity of patient data by making it homogeneous, interchangeable, global, and secure, with the intention of eventually integrating AI models into the radiotherapy workflow without raising ethical concerns.

Advances in outcome prediction using AI in radiation oncology

Another significant improvement that AI has brought to radiation oncology is the increased accuracy and refinement of outcome prediction models to anticipate both treatment response and potential complications and adverse effects.

The specialty of radiation oncology has long been focused on outcome prediction, even before the surge of AI and DL algorithms, primarily through tumor control probability and normal tissue complication probability [63]. Early models were able to use logistic regression to predict local tissue complications, such as esophagitis and pneumonitis in lung cancer treatment, rectal, bladder, and urinary fibrosis in prostate cancer, and mucositis and xerostomia from head and neck cancer radiation treatments [64]. However, most of these models prevalent in previous literature used 1D metrics such as the mean radiation dose that the tissue absorbed and did not consider differences between normal tissue versus neoplastic tissue response to radiation, which lacked the nuances of specific regions and cellular response, and therefore limited the potential accuracy of outcome prediction [63].

**Figure 4:**
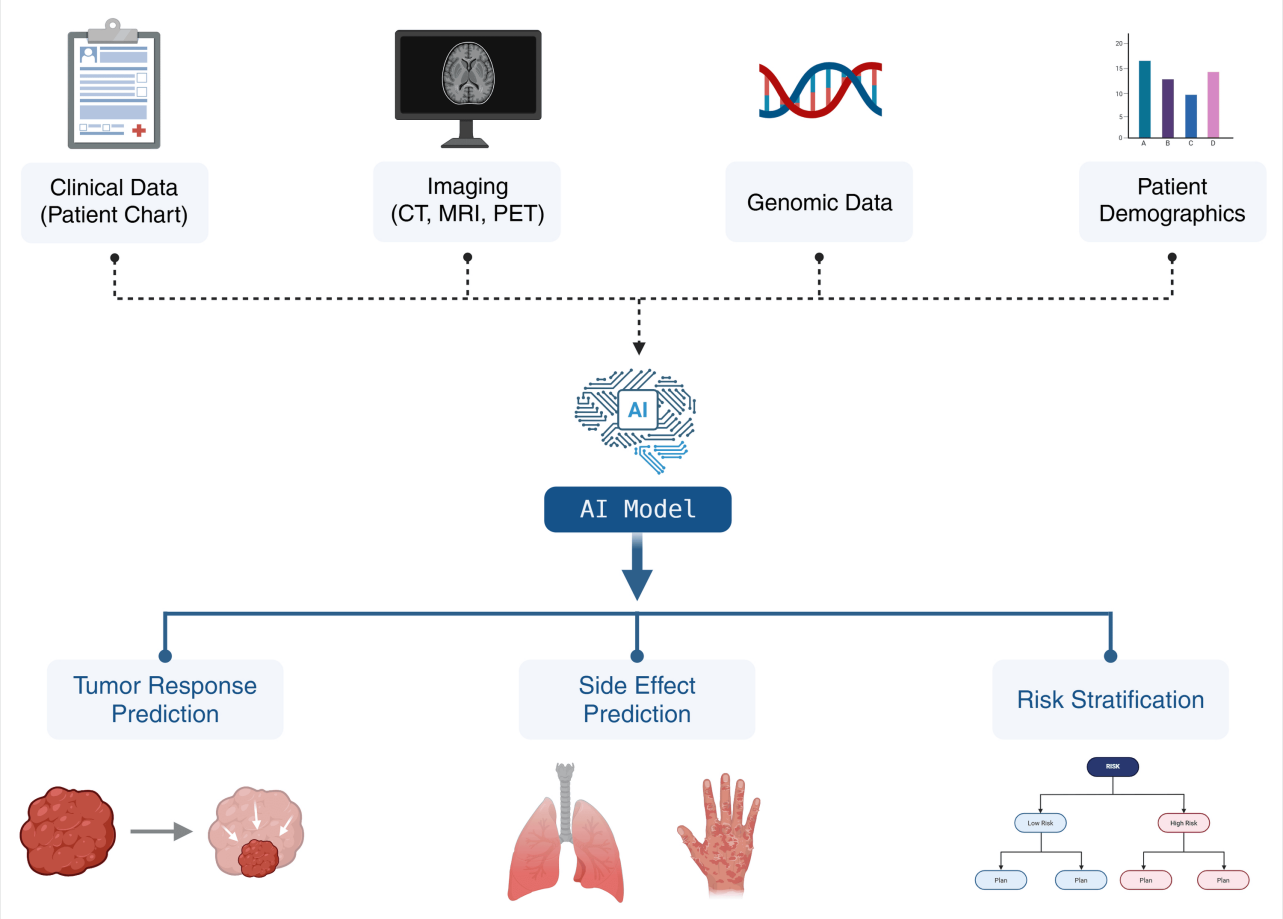
AI-powered model for outcome prediction [51,53,58,65] Created with BioRender by Fabeha Zafar (https://BioRender.com/yz3luj4)

Several variables influence clinical prediction models, including treatment selection, patient-specific anatomy, and spatial structure involvement, which makes the use of machine learning suitable to improve outcome prediction models, given that this method can detect different and previously unknown correlations that can be highlighted with DL approaches. Without this, problems in previous assumptions, assumed correlations, and simplifications could significantly influence and bias outcome predictions, limiting their accuracy [65]. This ability to integrate multiple, complex data sources into a single predictive framework is a core strength of modern AI-powered models (Figure 4).

Considering this, DL not only helps elucidate hidden correlations, but it can also incorporate patient-specific comorbidities and clinical factors such as performance status, imaging, genetic information, and age, providing individually tailored medicine and improving the reliability of predictive models [65].

One of the main methods used for this is supervised machine learning, where data is organized into a matrix in which rows correspond to patients, columns show specific features, and vectors indicate the outcome. By analyzing this information, the machine learning algorithm can find the correlations between these variables and outcomes, improving clinical knowledge and the accuracy of the algorithm for clinical prediction. However, many other approaches have their individual differences, advantages, and disadvantages. These include decision trees, support vector machines, Bayesian networks, ensemble methods, artificial neural networks, and more, depending on whether the data provided is structured (tables, numerical, and categorical) or unstructured (imaging, 3D contouring models, and histology slides). Each approach uses a model tailored for the data type [65].

Another important feature of these new models is their capability to be rapidly updated in response to changes and advances in treatment technologies, clinical variables, and correlations. As radiation oncology continues to evolve and new breakthroughs arise daily, these adaptive methods remain relevant over time and encourage further research, investment, and clinical validation, improving their predictive power and accuracy [64].

Although this scenario seems promising, the “black box” nature of AI models presents a challenge to our ability to learn from them because we cannot access an exact interpretation of how the conclusions were made. This alone is reason enough to maintain human reasoning and clinical knowledge as an essential part of the analysis of the new findings. Accordingly, correlations do not imply causation, and previous controversial results were found on AI usage before, such as spurious associations like screening colonoscopies being correlated to increased stroke risk, likely explained by the fact that these patients were older and had more comorbidities that increased the overall risk [66]. However, implementation of propensity matching and multivariate regression could help mitigate this concern. Until further studies are done, integrating AI models while simultaneously maintaining human clinical oversight is paramount to ensure treatment safety and continuous updates to our theoretical framework.

Challenges of using AI in radiation oncology

Despite the increased use of AI in the field of radiation oncology, several challenges lie ahead that must be addressed before AI applications can be more widely accepted.

Lack of Clear Clinical Utility

AI applications showed great promise in enhancing decision-making in radiation oncology and improving efficiency. However, one of the challenges with the use of AI models is that they do not provide any clear clinical utility. From a clinical standpoint, the added value of AI remains the biggest and most important question [9]. AI tools need to not only measure performance but also improve the aspects of the clinical setting. Therefore, clinical adoption is a key challenge and must be addressed to unlock the full potential of AI in clinical oncology [3].

Gap Between Efficiency and Clinical Benefit

**Figure 5:**
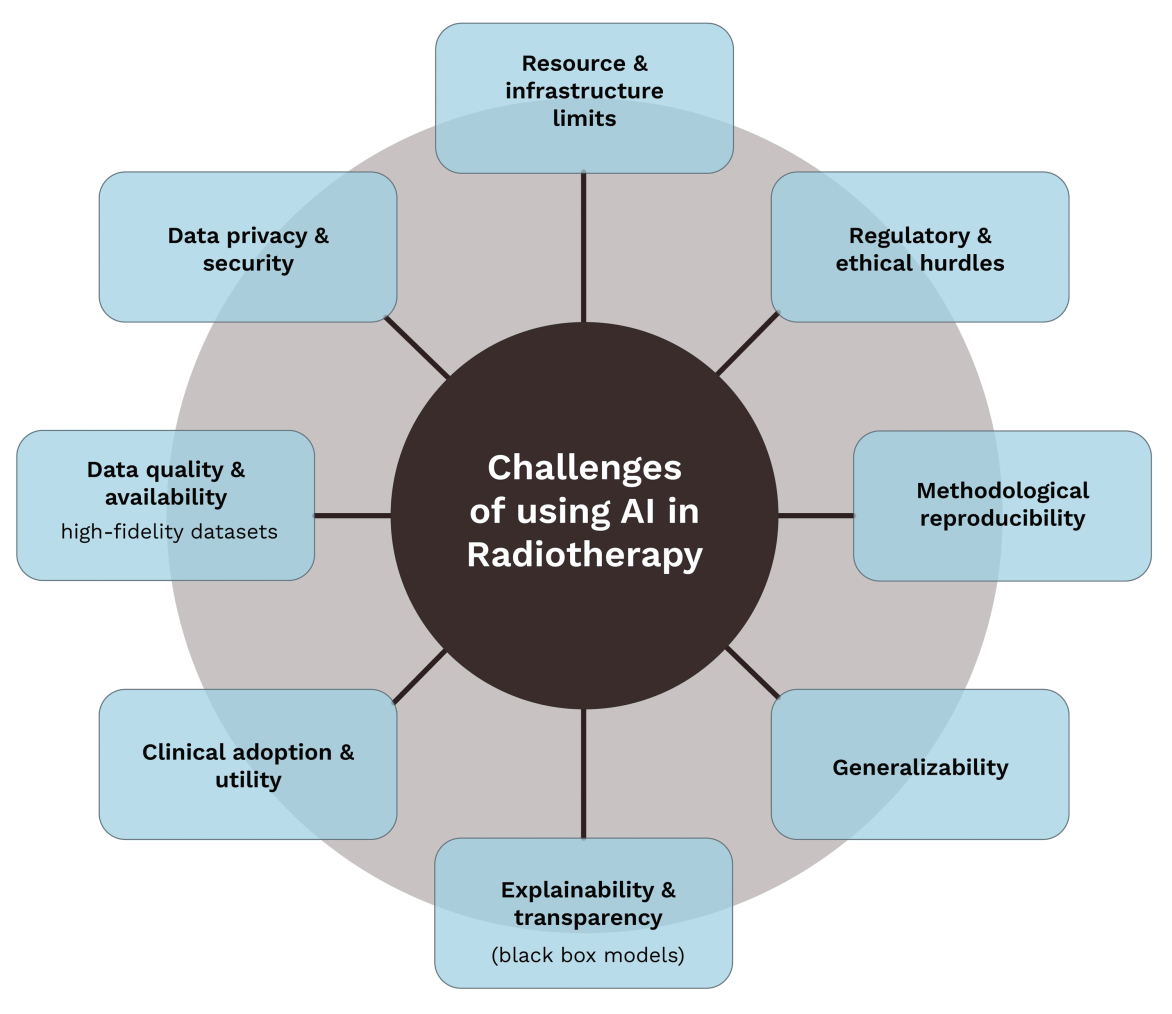
Challenges of using AI in radiotherapy [3,9,67] Original illustration created by co-author Ali R. Al Yousif

AI application in radiation oncology needs to provide clinical significance in improving the quality of life and improving the control rate and the survival rate. Most current AI-powered automated organ segmentation tools can increase efficiency when delineating target tumors and OAR during radiotherapy planning. AI tools have also proven to assist in decision-making, and randomized trials have shown that AI was able to effectively sort patients by risk and identify patients who require emergency room visits during radiotherapy. However, none of the AI tools currently being implemented showed any definitive improvement in the clinical value, such as decreasing recurrences, increasing survival, or improving patients’ quality of life. This opens doors for the development of AI tools that could impact clinical practice and lead to changes in patient outcomes, with future research focusing on their clinical utility. This gap between improving workflow efficiency and proving clinical benefit is one of the several challenges to the widespread adoption of AI in radiotherapy (Figure 5).

Data Quality and Reproducibility

A common point of concern with AI tools in radiation oncology is reproducibility and a lack of consistent standards in the generation of data. The result of the real-world application of AI is different from clinical research. The accuracy of AI models is strongly dependent on the nature of the input data; therefore, the most essential factor when developing an AI model is a high-quality database for algorithm training [3]. Future applications need to bridge the gap between research and clinical practice. This can be achieved through better study design and better data-sharing protocols between institutions.

Transparency and Reporting Standards

Data from AI research should adhere to the principles of the findable, accessible, interoperable, and reusable (FAIR) method to enhance transparency and testability of the data. In addition, the TRIPOD reporting guideline model should be implemented for developing and evaluating AI tools. This will further ensure transparency in reporting AI in health care. The need to use AI-repository modules such as modulhub.ai and AIMEBio is an essential step in order to support open access to training models for new research or studies that require reproducibility and generalizability [9].

Generalizability of AI Models

Another common challenge with the use of AI models is the generalizability of the data results because it may not accurately reflect the diverse patient population. Many AI models remain at the proof-of-concept stage due to a lack of external validation and generalizability [3]. It is important to understand that AI models will learn from site-specific artifacts that are included in the scan rather than learn from the clinical indicator. Therefore, to increase generalizability, access to multicenter data results with patients from different clinical settings, ethnicities, genders, geographic locations, and different institutional protocols should be implemented when developing an AI tool.

Equity and Representation in AI Development and Deployment

An area that warrants additional consideration is the representativeness of the datasets used to train and validate DL models in radiation oncology. Current data sources may not adequately capture the diversity of patient populations, particularly individuals from racial and ethnic minority groups or those of lower socioeconomic status or in less developed countries. This underrepresentation raises concerns about algorithmic bias, which could result in models that perform less effectively in precisely those populations already at higher risk for disparities in cancer outcomes. A related concern is that, even if high-performing AI models are developed, their adoption and integration into clinical practice may not occur uniformly across health care settings. Radiation oncology facilities serving under-resourced communities may face barriers to implementing such technologies, further widening the gap in access to cutting-edge care. Given that technological advancements in radiation oncology have been strongly correlated with improved patient outcomes [67], it is critical that equity considerations are deliberately built into both the development and dissemination of AI tools. Ensuring that models are trained on diverse, representative data and that strategies are in place to support equitable deployment will be essential to avoid exacerbating existing disparities.

Explainability and Physician Trust

The decrease in trust and acceptance of AI modules by physicians reflects the lack of understanding of the reasoning behind AI models’ predictions or decisions. The method “explainable AI” can help mitigate this issue by using saliency maps to represent the parts of an image that are most important to the model’s prediction. Using gradient-weighted class activation mapping provides a checkpoint to help confirm the location [9]. Transparency in training and testing data is a crucial step when developing AI models, and applications should be limited to a population with similar characteristics that were included during the training of the database.

Education and Training in AI

Future medical training needs to allocate more time to teaching AI tools to help bridge the knowledge gap about the understanding of AI reasoning behind model predictions. Additionally, peer-to-peer teaching in medical practice can further increase knowledge, acceptance, and confidence in AI tools.

Privacy and Data Security

Many AI tools are developed and owned by private entities, which means that these large corporations and institutions have access to, use, and control patients’ data [68]. This concern raises serious privacy challenges related to the security and use of these datasets. Appropriate regulations and safeguards must be implemented to maintain privacy and patient agency. These regulations should emphasize patient consent and agency. They should also recommend the use of sophisticated methods of data anonymization and security [68].

What does AI promise to offer in cancer care, and is AI trustworthy when assisting with radiation therapy?

Integrating AI into radiation oncology has been a transformative development, promising to enhance the precision and efficiency of cancer treatments. One of the most critical and time-consuming tasks in radiation therapy planning is delineating target volumes and OARs, commonly known as contouring. The advent of AI-based contouring tools, such as the Limbus^®^ AI software, can revolutionize this process by providing rapid and accurate delineations that could significantly impact treatment outcomes [69].

The future of AI in radiation oncology holds the promise of significant changes in patient care. The combination of AI with emerging technologies such as radiomics and genomics is expected to transform the field. Radiomics extracts numerous features from medical images, which AI then uses to predict disease progression and responses to treatment. Genomics provides insights into the genetic makeup of tumors, enabling more targeted and effective treatments. Explainable AI offers insights into how AI algorithms make decisions, helping clinicians understand and validate AI-based recommendations. Increasing transparency in AI systems will address concerns about bias and errors, encouraging wider adoption of AI in clinical settings. AI has the potential to improve clinical care quality while boosting efficiency. For example, studies show that AI can shorten the time needed for treatment planning and image analysis, allowing clinicians to focus more on patient care. AI-driven predictive analytics can identify patients at high risk for complications, enabling proactive interventions that improve outcomes. In addition, AI’s ability to learn and adapt from new data ensures that treatment strategies stay aligned with the latest medical knowledge and technological advances. In clinical practice, AI technologies can facilitate key advancements in radiation oncology, including improving diagnostic accuracy through AI-based image analysis, which enhances tumor detection and segmentation, thereby increasing the precision of radiation targeting and minimizing damage to healthy tissues; enabling real-time adaptive therapy by rapidly analyzing daily images and adjusting treatment plans on the spot; enhancing patient monitoring with wearable health devices combined with AI analytics, allowing continuous tracking of health indicators and timely interventions; and developing personalized treatment plans through the analysis of large datasets to identify patterns and predict individual responses, helping clinicians tailor therapies to each patient’s unique profile. Looking ahead, incorporating AI into radiation oncology requires careful attention to ethical, regulatory, and practical issues. Nonetheless, the potential benefits, such as better patient outcomes and improved operational efficiency, make it a valuable pursuit. Ongoing collaboration among multidisciplinary teams, the establishment of robust standards, and the continued development of AI will shape the future of radiation oncology, ultimately transforming patient care [70].

As AI technology advances, particularly in the health care sector, the ethical considerations associated with its use become increasingly complex and important. Farah et al.’s study emphasizes the criticality of AI performance, interpretability, and explainability in health technologies. Their research indicates that gaining a comprehensive understanding of how machine learning algorithms function and produce predictions is paramount for health care technology assessment agencies. The authors propose the development and implementation of tools to evaluate these aspects, thereby fostering a sense of accountability among stakeholders [71]. Fosch-Villaronga et al. examined the potentially reinforcing effects of gender bias in AI-based health care applications. They emphasize the importance of accounting for gender and sex differences in the development of medical algorithms, warning that the neglect of these factors could lead to misdiagnoses and potential discrimination. This research underscores the need to incorporate diversity and inclusion considerations into AI developments in health care to prevent exacerbating existing biases or creating new ones [72].

Many studies highlight the ethical considerations surrounding AI in health care, particularly regarding interpretability, accountability, and bias. They underscore the importance of ensuring that AI technologies are equitable, transparent, and complementary to human expertise, rather than functioning as replacements. Furthermore, they draw attention to the critical role of data governance and incentivization strategies in the development and application of AI technologies in the health care sector.

The use of AI in health care demands careful ethical consideration. There is a growing consensus that AI can significantly contribute to health care improvements. However, its application should not only address technical aspects but also the perspectives of health care professionals and patients. Key considerations include data security and privacy, the avoidance of bias, and ensuring that AI is responsive to diverse and local needs. Lastly, it is important that these technologies are developed and deployed responsibly and that they engender trust in those who use them [73]. Alongside the actual regulation and data protection, there are other legal implications of AI and its use, whether in health care or elsewhere. One of these is accountability. As soon as AI starts making autonomous decisions about diagnoses and treatments, moving beyond its role as merely a support tool, a problem arises as to whether its developer can be held accountable for its decisions.

Errors in AI appear mainly when confounding factors are correlated with pathologic entities in the training datasets rather than actual signs of disease. When an AI model provides a decision, it is based on the collected data, the algorithms it is based on, and the training data used to develop it. The reason that these decisions are unpredictable is twofold. On the one hand, AI models are excellent at identifying complex correlations within data, but they lack a true understanding of causation. On the other hand, this unpredictability stems from the inherent “black box” nature of AI models, where the specific reasoning pathway for a given decision remains opaque even to its developers.

AI will play an increasingly important part over the coming years in the relationship between doctors and patients, and it will need to be bound by core ethical principles, such as beneficence and respect for patients, which have guided clinicians throughout the history of medicine. We should remember, indeed, that the radiologist, as a physician, is much more than simply an interpreter of images. The duties of a practicing radiologist also include communication of findings, QA, quality improvement, education, interventional radiology procedures, policymaking, and many other tasks that cannot be performed by computer programs. The ability to provide a nuanced interpretation for complex findings, medical judgment, and the wisdom of an experienced radiologist is difficult to quantify and even more difficult to simulate with AI systems [74].

## Conclusions

As AI evolves, its application in medicine and specifically radiation therapy promises to revolutionize radiotherapy workflow from the very first image processed and to help establish a radiation-related prognosis in given patients. The introduction of AI in radiotherapy shows improvement in variability and time expenditure when compared to manual treatment planning steps, which directly impacts the efficiency of the workflow and its reproducibility. Improving efficiency is expected to lead to resource savings, a very valuable outcome for underserved populations with limited access to trained medical providers. In order to transition to AI-assisted radiotherapy, large datasets are needed to train the models. This represents a challenge due to the heterogeneity in available data and the need to preserve patients’ confidentiality. A key advantage of these developing models is the possibility of continuous updates in response to upcoming technology. As for patients’ clinical outcomes, more research needs to be conducted to establish the value of receiving AI-assisted radiotherapy.
